# Genome-wide DNA methylation profiling of non-small cell lung carcinomas

**DOI:** 10.1186/1756-8935-5-9

**Published:** 2012-06-22

**Authors:** Rejane Hughes Carvalho, Vanja Haberle, Jun Hou, Teus van Gent, Supat Thongjuea, Wilfred van IJcken, Christel Kockx, Rutger Brouwer, Erikjan Rijkers, Anieta Sieuwerts, John Foekens, Mirjam van Vroonhoven, Joachim Aerts, Frank Grosveld, Boris Lenhard, Sjaak Philipsen

**Affiliations:** 1 Department of Cell Biology, ErasmusMC, PO Box 2040, Rotterdam, CA, 3000, The Netherlands; 2 Center for Cancer Genomics, ErasmusMC, Rotterdam, The Netherlands; 3 Department of Biology and Computational Biology Unit, Uni BCCS, University of Bergen, Hoyteknologisenteret, Thormohlensgate 55, Bergen, N-5008, Norway; 4 Dutch Consortium for Systems Biology, ErasmusMC, Rotterdam, The Netherlands; 5 Center for Biomics, ErasmusMC, Rotterdam, The Netherlands; 6 Internal Oncology, ErasmusMC, Rotterdam, The Netherlands; 7 Department of Bioinformatics, ErasmusMC, Rotterdam, The Netherlands; 8 Department of Pulmonology, ErasmusMC, Rotterdam, The Netherlands; 9 Center for Biomedical Genetics, ErasmusMC, Rotterdam, The Netherlands

**Keywords:** DNA Methylation, Epigenetics, MethylCap, Next generation sequencing, Non-small cell lung Cancer

## Abstract

**Background:**

Non-small cell lung carcinoma (NSCLC) is a complex malignancy that owing to its heterogeneity and poor prognosis poses many challenges to diagnosis, prognosis and patient treatment. DNA methylation is an important mechanism of epigenetic regulation involved in normal development and cancer. It is a very stable and specific modification and therefore in principle a very suitable marker for epigenetic phenotyping of tumors. Here we present a genome-wide DNA methylation analysis of NSCLC samples and paired lung tissues, where we combine MethylCap and next generation sequencing (MethylCap-seq) to provide comprehensive DNA methylation maps of the tumor and paired lung samples. The MethylCap-seq data were validated by bisulfite sequencing and methyl-specific polymerase chain reaction of selected regions.

**Results:**

Analysis of the MethylCap-seq data revealed a strong positive correlation between replicate experiments and between paired tumor/lung samples. We identified 57 differentially methylated regions (DMRs) present in all NSCLC tumors analyzed by MethylCap-seq. While hypomethylated DMRs did not correlate to any particular functional category of genes, the hypermethylated DMRs were strongly associated with genes encoding transcriptional regulators. Furthermore, subtelomeric regions and satellite repeats were hypomethylated in the NSCLC samples. We also identified DMRs that were specific to two of the major subtypes of NSCLC, adenocarcinomas and squamous cell carcinomas.

**Conclusions:**

Collectively, we provide a resource containing genome-wide DNA methylation maps of NSCLC and their paired lung tissues, and comprehensive lists of known and novel DMRs and associated genes in NSCLC.

## Background

Non-small cell lung carcinoma (NSCLC) is a common malignancy characterized by a worldwide high incidence and low survival rate [1]. NSCLC is a heterogenic disease which is broadly classified into three major histopathological subtypes: adenocarcinoma (ADC), squamous cell carcinoma (SCC) and large cell carcinoma (LCC). This heterogeneity poses challenges for diagnosis and treatment, since each subtype presents with a distinctive prognosis [2] and the choice of therapeutic regimen is predominantly based on tumor subtype and staging parameters [3]. The development of personalized diagnostics and therapy is leading the way to a new era that may see us overcome some of the difficulties in treating complex diseases such as NSCLC.

In the past decade, comparative gene expression profiles of tumors have been extensively studied [4-6], yielding useful insights into the molecular hallmarks of carcinogenesis [7,8]. With the advent of next generation sequencing, genome-wide screening has become an attractive tool for profiling tumors versus lung tissues [7,9]. DNA methylation is a very stable epigenetic mark and next generation sequencing studies have recently shown that many genes are aberrantly methylated in cancer [10,11]. Tissue specific DNA methylation patterns are stabilized during embryonic development, and faithfully maintained through cell divisions [12-14]. Nevertheless, established methylation patterns can be reprogrammed, with tumor cells undergoing DNA demethylation and *de novo* methylation through mechanisms not yet completely understood. *CDKN2A* and *RASSF1* are examples of genes found to be aberrantly methylated in a wide variety of tumors [15-18], and epigenetic silencing of these genes has also been reported in NSCLC [19-22]. The great majority of DNA methylation studies are concentrated on the analysis of CpG islands located in the promoter areas of pre-selected genes. However, differentially methylated areas may be located within genes and at large distances from the nearest neighboring genes [23,24]. Although data on methylated genes in NSCLC are rapidly accumulating, unbiased data concerning specificity of the genome-wide distribution of methylated loci are still scarce.

In this study, we used Methyl-DNA Capture (MethylCap) and high-throughput sequencing (MethylCap-seq, [25]) to perform a genome-wide DNA methylation screening of NSCLC tumors and paired adjacent lung tissues. With this approach, we sought to identify genome-wide aberrant methylation patterns of NSCLC. Specific differentially methylated regions would be promising candidate molecular markers for non-invasive diagnostics using circulating tumor DNA, and increase the number of possible targets for epigenetic therapy.

## Results

### Methylation profiles in NSCLC-study outline

**Figure 1:**
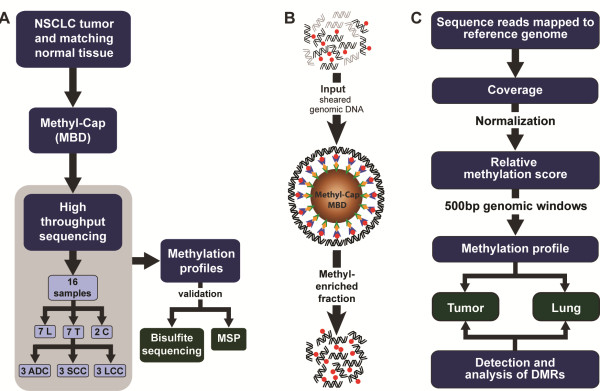
**Experimental design for profiling of DNA methylation patterns in non-small cell lung carcinoma.** (**A**) Overall view of the steps followed to generate the profiles (ADC: adenocarcinoma; LCC: large cell carcinoma; MBD: methyl-binding domain protein; N: Lung; SCC: squamous cell carcinoma; T: tumor), (**B**) MethylCap using methyl-binding domain proteins: Sheared genomic DNA is used as input fraction (methyl groups in red) and incubated with beads [26], coated with streptavidin (green)-biotin (yellow)) and linked to methyl-binding domain protein (blue) to capture methylated DNA. Captured fragments are subjected to high-throughput sequencing. (**C**) Summary of the bioinformatics approach used to generate the methylation profiles.

We performed genome-wide DNA methylation analysis of NSCLC using the MethylCap assay followed by high-throughput sequencing: MethylCap-seq (Figure 1A). We used DNA isolated from seven NSCLC tumors and paired lung tissues. Data regarding the samples used in this study can be found in Table 1 and Additional file 1. As controls, we prepared fully methylated and fully unmethylated genomic DNA. The DNA samples were sheared and then enriched for methylated DNA using the MethylCap procedure [25]. This is based on the capture of methylated DNA by biotinylated methyl-binding domain protein (MBD), which is then retrieved by binding to streptavidin-coated beads (Figure 1B). The recovered DNA was directly sequenced using the Illumina Genome Analyzer IIx next generation sequencing platform. For each DNA sample, we performed two independent enrichment procedures and sequence runs. Total numbers of sequence reads, mapped reads and unique reads for each sample are represented in Additional file 2. For th identification of differentially methylated regions (DMRs) we employed a rigorous normalization procedure and a variety of bioinformatics tools (Figure 1C). We used MethylCap-seq data obtained from artificially prepared fully unmethylated and fully methylated DNA samples for normalization of the data and assignment of DMRs. The bioinformatics approach is described in detail in Additional file 3 and Additional file 4. Relative methylation scores were used to build an individual methylation profile for each sample. Subsequently, we compared the profiles to identify highly significant DMRs between tumors and paired lung tissues, and between the subtypes of tumors. Finally, we validated the MethylCap-seq results by bisulfite sequencing and methyl-specific PCR of selected DMRs.

**Table 1:** Data from patients used for MethylCap-seq and bisulfite sequencing validation

**Sample ID**	**Tissue type**	**Patient ID**	**Stage**	**Overall survival (month)**	**Survival status**	**Gender**	**Age at diagnosis**	**Ethnicity**
2213 N	healthy	SCC1	IIB	2,43	Alive	M	54,54	Caucasian
2214 T	SCC							
2235 N	healthy	SCC2	IIB	51,23	Deceased	M	73,73	Caucasian
2236 T	SCC							
2245 N	healthy	ADC1	IB	12,57	Alive	F	66,26	Caucasian
2246 T	ADC							
2255 N	healthy	ADC2	IA	98,93	Deceased	F	54,26	Caucasian
2256 T	ADC							
2257 N	healthy	ADC3	N/A	N/A	N/A	M	78,96	Caucasian
2258 T	ADC							
2261 N	healthy	SCC3	IIB	6,77	Alive	M	70,03	Caucasian
2262 T	SCC							
22he	healthy	LCC1	IB	35,73	Deceased	F	56,58	Caucasian
22tu	LCC							

ADC: adenocarcinoma; LCC: large cell carcinoma; N/A: not applicable; SCC: Squamous cell carcinoma.

### Global analysis of genome-wide methylation patterns of NSCLC

**Figure 2:**
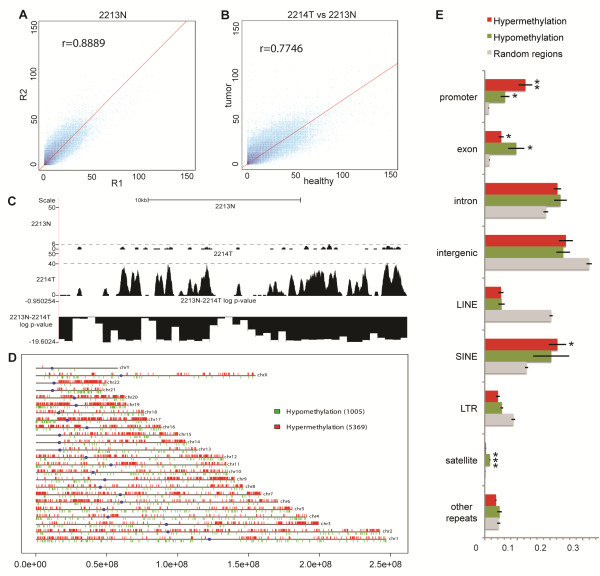
**Global analysis of DNA methylation patterns in non-small cell carcinoma.** (**A**) Correlation between experimental replicates. Each point represents the raw methylation signal (mean coverage in 10 bp bins). Density of points (log10 scale) is shown in different shades of blue. Pearson’s correlation coefficient is denoted in the top left corner of each scatter plot. (**B**) Correlation between tumor (x axis) and paired lung tissue sample (y axis). (**C**) Raw reads from a lung sample (above panel) and its matching tumor sample (middle panel) in the position chr2:176,716,200 to 176,738,910 viewed in the UCSC genome browser; normalized *P*-value is depicted in the bottom panel. Y-axis depicts the number of sequence reads in each region per sample. Dashed line represents the maximum number of reads (highest peak) in each sample; (**D**) Representation of the distribution of hypomethylated (green) and hypermethylated (red) regions across chromosomes in a tumor versus paired lung sample. (**E**) Composition of hypo- and hypermethylated regions in comparison to randomly sampled regions across the genome. Bars indicate the mean proportions of regions covered by each distinct genomic feature. Error bars denote standard error of the mean. Promoters were defined as regions ± 1 kb from all Ensembl transcription start site. Distinct classes of repeats were retrieved from the UCSC Table Browser (RepeatMasker table for hg18). Repeats were excluded from all subsequent features. Random regions were sampled across the genome independently seven times and each time their number was matched to the number of differentially methylated region in one sample pair. Statistical testing was done against random regions using one-tailed Student’s t-test (*P-≤0.01; **P-≤0.001; ***p≤0.0001).

To assess the reproducibility of the MethylCap-seq procedure, we first performed a self/self comparison of the replicate experiments. This yielded an average Pearson’s correlation coefficient of 0.89 (Figure 2A and Additional file 5), indicating excellent reproducibility between independent experiments. Next, we compared methylation signal in tumors with matched healthy lung tissues; the average correlation coefficient between the two was 0.83, indicating a generally high similarity in the methylation patterns of the matched tumor and lung samples (Figure 2B and Additional file 6). Collectively, these analyses establish the high reproducibility and specificity of the MethylCap-seq procedure that we used. To allow visual inspection of the methylation signal and DMRs, raw reads and log *P*-values of differential methylation were uploaded in the University of California, Santa Cruz (UCSC) Genome browser, where DMRs can be visually inspected (Figure 2C). To focus on the most significant DMRs, we used a very stringent *P*-value threshold of 10^-18^, corresponding to a false discovery rate ≤ 10^-15^ ( Additional file 7). Non-repetitive regions with *P*-value ≤10^-18^ were projected onto the genome and visualized as genome-wide maps of differential methylation (Figure 2D and Additional file 8). This revealed that in the non-repetitive fraction of the genome, tumors displayed higher overall hypermethylation than the matching lung tissues. This is consistent with the notion that *de novo* methylation and hypermethylation of promoter CpG islands are associated with carcinogenesis [27].

The DMRs were analyzed according to general hallmarks of their genomic localization (Figure 2E). Compared with the general distribution of randomly sampled regions across the genome (label “Random regions”), we found that promoter areas were particularly enriched among the DMRs, both hypo- (*P*=5.7 × 10^-3^) and hypermethylated (*p*=5.3 × 10^-4^). Although we removed most signals from repetitive regions by keeping only uniquely mapped reads, the signals from their uniquely mappable reads and flanking regions were still above background and allowed us to test for differential methylation. This revealed that some specific repeat classes such as LINE and satellites were also differentially represented in the DMRs, with satellite repeats being particularly hypomethylated in tumors when compared with lung tissues (*P*=1.3 × 10^-5^). By contrast, LINE repeats are relatively underrepresented in the DMRs (hypo and hypermethylation), indicating that the methylation status of the bulk of the LINE elements was similar between the tumors and paired lung counterparts.

### Differentially methylated regions in NSCLC

**Figure 3:**
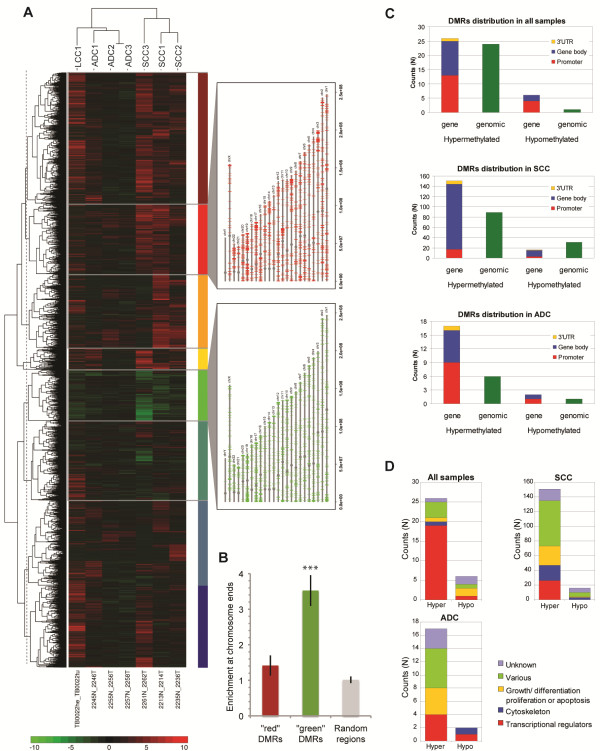
**Differentially methylated region in non-small cell carcinoma versus paired lung tissue samples.** (**A**) Heatmap of 14,742 most significant DMRs, picturing regions with mostly hypermethylated (top), mostly hypomethylated (middle), and mixes (bottom). Color bar at the bottom represents the log ratio of the normalized signal in tumor versus. lung (red= hypermethylation, green= hypomethylation). Dashed line represents cut in the dendogram, generating nine groups of DMRs. The DMRs from the two most distinctive groups are depicted on the chromosomes. Red: cluster containing regions hypermethylated in all samples; green: cluster containing regions hypomethylated in all samples. (**B**) Bar plot showing mean enrichment of DMRs at chromosome ends calculated as ratio between proportion of 1 Mbp region at chromosome ends and total proportion of each chromosome covered by DMRs. Error bars show standard error of mean of all chromosomes. Statistical testing was done against random regions using one-tailed Student’s t-test (***P≤0.0001). (**C**) DMRs distribution relative to gene position. Number of hyper- and hypomethylated genes and regions (outside gene area), and their distribution as in promoter, gene body and 3′ UTR (top panel: all seven samples; middle panel: squamous cell carcinomas, bottom panel: adenocarcinoma). (**D**): Gene function distribution of the differentially methylated genes showing number of hyper- and hypomethylated genes relative to gene classes or function. DMR: differentially methylated regions; UTR: untranslated region.

We observed a total of 14,742 DMRs in the seven NSCLC tumor samples ( Additional file 9), that is regions found to be differentially methylated in at least one tumor when compared with its lung counterpart with a two-fold change difference in methylation signal. Performing unsupervised cluster analysis with these DMRs revealed that the samples clustered according to the histological subtype of the tumors (Figure 3). In particular, hypermethylated DMRs were overrepresented in SCC samples. The dendogram of the DMRs indicated the presence of nine major subgroups of DMRs. Two of these were composed of generally hypermethylated (red) or hypomethylated (green) DMRs. These were plotted on the chromosomes, revealing that the ‘red’ DMRs were scattered throughout the chromosomes. By contrast, the ‘green’ DMRs displayed a more localized distribution towards the chromosome ends (Figure 3B), showing on average 3.5^-^times greater enrichment than random regions (*P*=6.8 × 10^-6^). Subtelomeric hypomethylation has been found to be associated with risk of developing cancer [28].

We found 57 DMRs present in all tumor samples, of which 50 were hypermethylated and 7 were hypomethylated. An example of two DMRs in all samples, including the replicate experiment can be found in Additional file 10. We also analyzed DMRs in relation to NSCLC subtypes. We found 287 DMRs unique for SCC and 26 DMRs unique for ADC (Additioonal File 11). These DMRs were classified by hyper- and hypomethylation and their genomic locations relative to genes (Figure 3B). We found that the ADC-specific DMRs had a genomic distribution similar to that observed for the DMRs present in all seven tumors, while for SCCs, DMRs within genes were concentrated in the gene body. SCC also presented considerably more subgroup-specific DMRs than ADC and ‘All-sample’; this could be explained by the notion that SCC is a relatively homogeneous tumor type.

For the DMRs that fall in gene areas, we analyzed which types of genes were present in each of the three groups (Figure 3C and Additional file 11). We found that for the DMRs present in all tumor samples, more than 75% of the associated genes belonged to the class of transcriptional regulators. For SCC-specific DMRs, we observed a considerable heterogeneity in the functional categories of associated genes. Nevertheless, there was an overrepresentation of genes involved in transcriptional regulation, organization of the cytoskeleton and cell cycle regulation. We note that while some of the DMRs were associated with genes that had been previously reported to be differentially methylated in lung tumors, such as *APCCDH13CDKN2ADAPKhMLH1HOX genes, OTX1HOX2* and many others [22,29-32], we also found DMRs which, to the best of our knowledge, have not been previously reported to be associated with methylation in NSCLC or other types of cancer.

### Validation of differentially methylated regions in NSCLC by bisulfite sequencing

**Figure 4:**
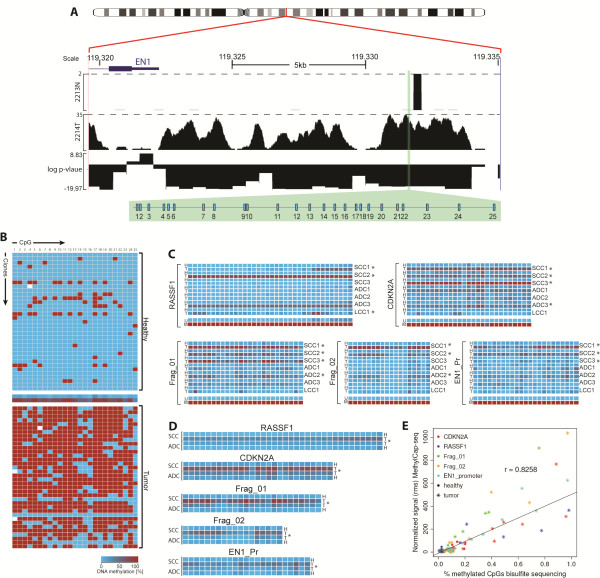
**Bisulfite sequencing validation for MethylCap-Seq.** (**A**) Sequence reads for the Frag_01 (genomic region located at position Ch2: 119,331,343 to 119,331,692) in a tumor (2214 T) and matching lung tissue (2213 N), plotted in the Genome Browser, showing the distribution of the 25 CpGs contained in the fragment highlighted in green. Dashed lines represent the highest number of reads in 2213 N and 2214 T. (**B**) Methylation status of each CpG in all 36 individually sequenced clones in the same samples and fragment shown in Figure 4A. The middle row represents the average of methylation in all clones per CpG position. (**C**) Average of methylation of all clones sequenced per patient in each fragment (M: control totally methylated DNA; N:lung; T:tumor; U:control totally unmethylated DNA). (**D**) Average of the methylation status of the sum of all clones, grouped per histological subtype; comparison betweenadenocarcinoma and squamous cell carcinoma in all fragments were statistically significant. (**E**) Correlation between normalized methylation signals from MethylCap-seq and CpG methylation from bisulfite sequence. Different regions are shown in different colors; lung samples are marked by dots and tumors by stars. Pearson’s correlation coefficient is denoted above the linear regression curve. *P≤0.001.

To validate the results obtained with MethylCap-seq, we selected five fragments for analysis by bisulfite sequencing. We chose *CDKN2A* and *RASSF1* since hypermethylation of these genes has been widely reported in a variety of cancers including NSCLC [15,21,22], and the *EN1* promoter region since hypermethylation of *EN1* had been previously reported in a lung tumor cell line [29] but not yet in primary NSCLC tumors. Since we observed that the region upstream of *EN1* was highly hypermethylated in the tumors and involved a region of more than 15 kbp (Figure 4A), we selected two extra fragments within this region, in addition to the *EN1* promoter. The other two fragments were located at positions chr2:119,331,343 to 119,331,692 and chr2:119,328,097 to 119,328,400 and we named them Frag_01 and Frag_02, respectively. Selection of the fragments for bisulfite sequencing analysis was based on the MethylCap-seq data (Figure 4).

To assure sufficient depth of coverage for quantitative analysis, we sequenced 36 individual clones from each fragment and each bisulfite-converted DNA sample. Figure 4B shows an example of the methylation status for one fragment of a paired sample. The methylation status of the five fragments in all seven paired NSCLC/lung tissue samples is summarized in Figure 4C; full data for all samples is available in Additional files 12 and 13. We also examined potential differences in methylation according to tumor histology. To perform this analysis we grouped the samples in four categories: all clones from the lung tissues of patients (ADC_N and SCC_N) and clones from tumors (ADC_T and SCC_T), (Figure 4D and Additional file 14). We observed a statistically significant difference (*P≤*0.0001), not only between tumors and lung tissues, but also between ADC and SCC for all fragments analyzed by bisulfite sequencing. This identifies these fragments as candidate markers for differentiating tumors versus paired lung tissues, and also between tumor subtypes. Moreover this finding validates the MethylCap-seq data, since the methylation status determined by bisulfite sequencing correlated quantitatively with the MethylCap-seq data (Figure 4E).

### Screening by methylation-specific PCR verifies methylation differences between tumor and paired lung tissues, and between tumor subtypes

**Figure 5:**
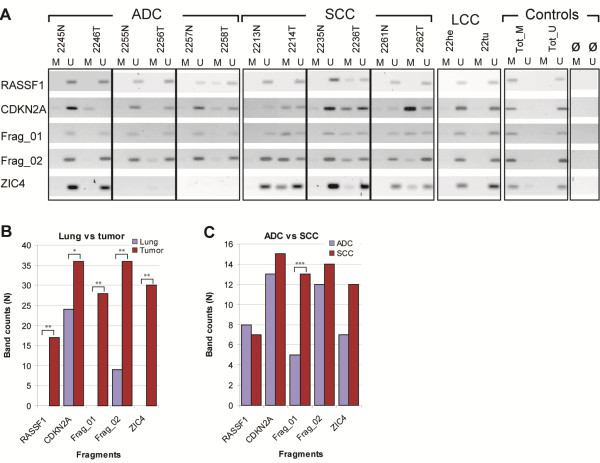
**Methylation-specific polymerase chain reaction screening in 48 tumors and matching lung tissues.** (**A**) Cropped gel images for the seven patients used for MethylCap-seq, grouped by histological subtype and controls (M: “methylated primer set”; N: lung; T: tumor; Tot_M: totally methylated control samples; Tot_U: totally unmethylated control sample; U: unmethylated primer set; ø: blank/water). (**B**) Histogram of the methylation status in lung samples and tumor samples in all 48 patients in the five fragments analyzed. (**C**) Histogram of the methylation status per histological subtype (statistics were calculated with Fisher’s exact test, two-tailed *p*-values: *P≤0.02; **P≤0.001 and ***P≤0.01).

To screen the methylation status of selected DMRs in a larger sample set we used methylation-specific PCR (MSP) [33]. Here, we selected RASSF1, CDKN2A, Frag_01 and Frag_02 because they were the most significant differentially methylated fragments in the bisulphite sequencing experiments. We added ZIC4 because it was one of the DMRs not yet reported as methylated in NSCLC and presented a high difference in relative methylation score between lung and tumor samples. We screened 96 samples (48 NSCLC and paired lung samples), and used totally methylated and totally unmethylated DNA samples as controls for the specificity of the MSP assays. Results for the seven paired samples used in MethylCap-seq are displayed in Figure 5. Data for all 96 samples are summarized in Table 2 and shown in Additional file 15.

**Table 2:** Frequency of methylation by methylation-specific polymerase chain reaction in the 96 samples (48 tumor and matching lung tissue) in five fragments

	**CDKN2A**			**Frag_01**			**Frag_02**			**RASSF1**			**ZIC4**		
	count (n)	Sensitivity (%)	Specificity (%)	Count (n)	Sensitivity (%)	Specificity (%)	Count (n)	Sensitivity (%)	Specificity (%)	Count (n)	Sensitivity (%)	Specificity (%)	Count (n)	Sensitivity (%)	Specificity (%)
Lung	24/48	75	27	0/47	62	100	9/48	75	56	0/41	42	100	0/39	77	100
Tumor	36/48*			29/47**			36/48**			17/41**			30/39**		
Adenocarcinoma	13/17	76.5	46.4	5/16	31.3	27.7	12/17	70.6	46.1	8/15	53.3	53.3	7/13	53.9	36.8
Squamous cell carcinoma	15/16	93.8	53.6	13/16	81.3**	72.2	14/16	87.5	53.9	7/16	43.8	46.7	12/16	75.0	63.2

Sensitivity is calculated using the ratio between methylated (amplified) sample and the total amount of samples analyzed per fragment; specificity is the ratio of the difference between amplification in tumor and in the matching lung sample (or adenocarcinoma versus squamous cell carcinoma). Statistical significance of the difference between control lung samples and tumors, and between adenocarcinomas and squamous cell carcionmas were calculated using chi-square. *P<0.02; **P0.0001.

MSP is a more qualitative approach than bisulfite sequencing. In MSP, selective amplification depends on the methylation status of only the CpGs present in the designed primers, which were two or three CpGs depending on the fragment. For each methylated (M) primer set, amplification of the fragment is dependent on methylation of the CpGs that are present in the sequence targeted by the “M” primer; while each unmethylated (U) primer set requires unmethylated CpGs in the referred sequence for amplification. In the 48 paired samples analyzed, *CDKN2A* showed frequent amplification with the M primer set in lung samples (24 out of 48), although the amplified bands were often of low intensity. Tumors had a much larger number displaying amplification with both M and U primer sets. Of the 47 tumors, 36 displayed amplification with the M primer set, and two of the tumors displayed amplification only with the M primer set. For RASSF1 and ZIC4, 41 paired samples were available. Both fragments differentiated between lung and tumor samples, whereas no methylation was detected in lung samples. ZIC4 showed methylation in more than 70% of the tumors, which is much higher than the 41% found for RASSF1. The findings for RASSF1 are in accordance with other studies [18]. Also, Frag_01 presented no amplification with the M primer set in the lung samples, in the Frag_02 M primer set only 5 out of 48 were amplified. In tumors, we observed fragment amplification in 29out of 48 samples for Frag_01 and 36out of 48 samples for Frag_02 with the M primer sets. Differences in methylation status were significant in tumors versus lung samples for all 5 fragments, with a *p*-value < 0.02 for CDKN2A and *P* < 0.0001 for the other four fragments (Figure 5B). Frag_01, Frag_02 and ZIC4 present a much lower *P*-value, indicating they may be better tumor markers than CDKN2A and RASSF1. We also observed that significant differences between SCC and ADC were only found for Frag_01 (Figure 5C). In conclusion, the MSP results validate the MethylCap-seq data, since the results obtained for all five fragments were consistent with the significant DMRs in all tumors, and Frag_01 appeared as part of the SCC-specific DMRs in the MethylCap-seq data.

## Discussion

To date, genome-wide methylation studies in NSCLC have concentrated on specific areas of the genome [30], promoter regions [34-36] or sets of pre-selected candidate genes [19,37]. Recently Kwon *et al*. [38] published a report on genome-wide analysis of DNA methylation in NSCLC. However, they also concentrate the analysis on a selection of candidate genes. In our study, we performed an unbiased genome-wide DNA methylation analysis of seven individual patients with NSCLC and their adjacent lung tissues. We combined MethylCap with high-throughput sequencing (MethylCap-seq) to draw detailed methylome maps of NSCLC tumors and paired lung tissues. The replicate experiments showed that the depth of coverage generated by MethylCap-seq was sufficient to capture the methylation status of the entire genome. Moreover, there was a very high degree of correlation between the replicate experiments, demonstrating the robustness of the MethylCap-seq approach. As controls for normalization and validation purposes we made use of fully methylated and fully unmethylated DNA samples. The MethylCap-seq data of these controls provide a useful resource for benchmarking of the fully methylated *versus* fully unmethylated status of any particular fragment in the genome. Using the MEDIPS computational tool [39], we generated methylation profiles of the seven paired NSCLC/lung cases which can be viewed in the genome browsers. This broad view of DNA methylation in NSCLC will provide new opportunities for the identification of specific epigenetic markers of NSCLC that could, for instance, be used for early detection of the disease.

The occurrence of methylated CpGs in the fragments selected for bisulfite sequencing showed a high quantitative correlation with the reads found by MethylCap-seq in the same area. In conjunction with the MethylCap-seq data on the fully methylated and fully unmethylated controls, this demonstrates that MethylCap-seq yields reliable quantitative information on the methylation status of the fragments in a particular DNA sample. This is important considering that the tumor cell content of samples to be analyzed will be variable. Provided that sufficient sequencing depth is achieved, it should be possible to use MethylCap-seq for the identification of tumor-specific hypermethylated DNA regions in samples containing only a minority of tumor cells, and possibly in circulating tumor DNA isolated from serum samples. The results obtained with MSP are qualitative in nature, but this PCR-based approach is very sensitive and may therefore be used to detect tumor-specific hypermethylated DNA fragments in serum DNA. As a first step in this direction, we used MSP to investigate the presence of five DMRs in a larger set of paired samples. The results confirmed the MethylCap-seq data and demonstrated that these DMRs are frequently hypermethylated in NSCLC. In particular, the Frag_01 fragment was found to be hypermethylated in 13 out of 16 SCC cases and in none of the ADC, suggesting that this might be a useful marker for this type of NSCLC, and ZIC4 showed 100% specificity for tumors and higher sensitivity (>70%) than RASSF1. In addition, *CDKN2A* amplification with the M primer was often observed in lung tissue samples, although at much lower amplification levels when compared with tumor samples. This could reflect an early event in the methylation status of *CDKN2A* in tumorigenesis.

The mapped reads point to differentially methylated areas across the genome, with some marked preferences for hypermethylation or hypomethylation in certain genomic regions. There is a strong correlation between CpG islands and hypermethylation in tumors, with the most significantly hypermethylated regions associated with promoter regions of genes. As previously reported by many groups [40-43], gene promoters are a target of methylation as an epigenetic regulatory mechanism. However, our data also reveal a high degree of hypermethylation outside promoter areas at intergenic regions, and at long distances from genes. This indicates that patterns of DNA methylation may play a role not only by silencing promoters of key tumor suppressor genes, but also by regulating gene expression in a more complex manner through distant regulatory elements such as insulators and enhancers.

We found that satellite regions were particularly hypomethylated in the NSCLC tumors when compared with lung tissues. Recently, Ting et al. [44] have shown that overexpression of satellite repeats is associated with different types of cancer. The observed hypomethylation of these regions in NSCLC would be compatible with their results. We also found that hypomethylated regions are often present in subtelomeric regions, in agreement with the observation that hypomethylation is more frequently located near chromosome ends in lung cancer [45]. While CpG islands and genes are present in the subtelomeric regions of chromosomes, the impact of subtelomeric hypomethylation on the regulation of these genes is currently unclear.

In an alternative approach, we selected the most significant DMRs in the individual NSCLC cases (14,742) and performed unsupervised cluster analysis of the hyper- and hypomethylated regions in the tumor samples. We observed that the samples clustered according to their histological classification. This indicates that there are DMRs that could not only be used as NSCLC markers but also as markers for histological classification of NSCLC tumors. Subsequently we searched for the specific areas that were differentially methylated in all tumors when compared with the paired lung tissues, and specific for the subtypes. We observed that SCCs displayed more DMRs than did ADCs did. This observation could be explained by the heterogeneity of ADCs, which would result in fewer DMRs shared by all three ADC samples. The contrary is seen in SCC, which is a more homogeneous type of NSCLC. This characteristic has been observed previously in gene expression profiling studies [4,46].

Remarkably, the majority (>75%) of the hypermethylated genes present in all seven samples encoded for transcription factors, while the shared hypomethylated genes were spread among many different functional categories. Transcription factors play a central role in maintaining or modifying cell fate, either in normal processes such as development and cell differentiation, or in cancer. Mechanisms of epigenetic regulation via DNA methylation are not yet completely understood; several factors such as DNA methyltransferases, chromatin remodelling proteins and DNA-binding transcription factors are involved [47,48]. Therefore, any perturbation that leads to a decrease in the expression status of those factors may disrupt important pathways for maintenance of the differentiated cellular state. Aberrant methylation can change chromatin structure, rendering DNA binding sites accessible or inaccessible to transcription factors leading to activation or silencing of genes important in cell differentiation and maintenance. It has been recently shown that hypermethylated genes found in lung tumors are associated with cellular morphogenetic differentiation [49]. These cellular mechanisms are orchestrated by transcriptional regulators; therefore changes in the methylation status of their binding sites may disrupt those processes thus contributing to oncogenesis.

## Conclusion

Based on the MethylCap-seq data, we generated a list of candidate DMRs and associated genes specific for NSCLC and its subtypes (Table 3). Several of these DMR-associated genes are known to be hypermethylated in NSCLC and other types of cancer, strongly supporting the validity of the data set reported here. For instance, the observed methylation rates of *RASSF1* were in agreement with previously reported results [18]. In the MetylCap-seq data, hypermethylation of the genomic region where Frag_01 is located was most significant for the SCC samples. This was supported by the bisulfite sequencing and MSP validation experiments, where this fragment showed significantly more hypermethylation in SCCs when compared with ADCs or LCCs. In conclusion, this list of candidate DMR markers can be used to develop sensitive biological markers for NSCLC, which may enable non-invasive diagnosis and early detection of the disease, and potentially allow histological classification. Collectively, we provide a resource containing genome-wide DNA methylation maps of NSCLC and paired lung tissues, and comprehensive lists of DMRs and associated genes in NSCLC.

**Table 3:** List of most differentially methylated genes in non-small cell carcinoma

	**Gene**	**Log**_**2**_**ratio**	**Gene class/type/ biological function**	**Gene name**	**HGNC ID**
Common in all tumors	*BARX1*	4.8	transcription regulator	BARX homeobox 1	HGNC:955
	*PAX9*	4.3	transcription regulator	paired box 9	HGNC:8623
	*OTX1*	4.0	transcription regulator	orthodenticle homeobox 1	HGNC:8521
	*NPR3*	3.8	G-protein coupled receptor	natriuretic peptide receptor C/guanylate cyclase C (atrionatriuretic peptide receptor C)	HGNC:7945
	*FGF12*	3.7	growth factor	fibroblast growth factor 12	HGNC:3668
	*ONECUT2*	3.6	transcription regulator	one cut homeobox 2	HGNC:8139
	*PRDM14*	3.5	transcription regulator	PR domain containing 14	HGNC:14001
	*RAX*	3.5	transcription regulator	retina and anterior neural fold homeobox	HGNC:18662
	*SHOX2*	3.3	transcription regulator	short stature homeobox 2	HGNC:10854
	*DMRTA2*	3.1	transcription regulator	DMRT-like family A2	HGNC:13908
	*FER1L4*	3.1	unknown	fer-1-like 4 (*Caenorhabditis*. elegans) pseudogene	HGNC:15801
	*SIX6*	3.1	transcription regulator	SIX homeobox 6	HGNC:10892
	*GATA3*	3	transcription regulator	GATA binding protein 3	HGNC:4172
	*SKOR1*	3	transcription regulator	SKI familytranscriptional corepressor 1	HGNC:21326
	*HOXA9*	3	transcription regulator	homeobox A9	HGNC:5109
	*SALL1*	2.9	transcription regulator	sal-like 1 (Drosophila)	HGNC:10524
	*IRX2*	2.7	transcription regulator	iroquois homeobox 2	HGNC:14359
	*GRIK2*	2.7	ion channel	glutamate receptor, ionotropic, kainate 2	HGNC:4580
	*SATB2*	2.6	transcription regulator	SATB homeobox 2	HGNC:21637
	*MEIS1*	2.5	transcription regulator	Meis homeobox 1	HGNC:7000
	VAX1	2.4	transcription regulator	ventral anterior homeobox 1	HGNC:12660
	TBX15	2.3	transcription regulator	T-box 15	HGNC:11594
	*CNTD2*	−2.4	unknown	cyclin N-terminal domain containing 2	HGNC:25805
	*ZMYND10*	−2.6	unknown	zinc finger, MYND-type containing 10	HGNC:19412
	*MYC*	−2.9	transcription regulator	v-myc myelocytomatosis viral oncogene homolog (avian)	HGNC:7553
	*TSPAN9*	−2.9	Plasma Membrane	tetraspanin 9	HGNC:21640
	*NAV1*	−3.1	unknown	neuron navigator 1	HGNC:15989
	*CPEB3*	−3.4	unknown	cytoplasmic polyadenylation element binding protein 3	HGNC:21746
Unique for squamous cell carcinomas	*TRAPPC9*	6.5	cell differentiation	trafficking protein particle complex 9	HGNC:30832
	*ABHD2*	6.3	hydrolase	abhydrolase domain containing 2	HGNC:18717
	*CTNND1*	6.2	transcrption regulator	catenin (cadherin-associated protein), delta 1	HGNC:2515
	*HIST1H2BB*	6.2	histone protein	histone cluster 1, H2bb	HGNC:4751
	*EMP1*	5.8	protein binding	epithelial membrane protein 1	HGNC:3333
	*TBL1XR1*	5.6	transcription regulator	transducin (beta)-like 1 X-linked receptor 1	HGNC:29529
	*NXPH1*	5.6	protein binding	neurexophilin 1	HGNC:20693
	*ZIC4*	5.6	transcription regulator	Zic family member 4	HGNC:20393
	*AOAH*	5.4	hydrolase	acyloxyacyl hydrolase (neutrophil)	HGNC:548
	*ACTN4*	5.4	transporter	actinin, alpha 4	HGNC:166
	*C1orf21*	5.2	unknown	chromosome 1 open reading frame 21	HGNC:15494
	*PACSIN2*	5.2	transporter	protein kinase C and casein kinase substrate in neurons 2	HGNC:8571
	*PMM2*	5.2	isomerase	phosphomannomutase 2	HGNC:9115
	*DOT1L*	5.1	histone methyltransferase	DOT1-like, histone H3 methyltransferase (*Sacchormyces*. *cerevisiae*)	HGNC:24948
	*WWP2*	5	e3 ubiquitin-protein ligase	WW domain containing E3 ubiquitin protein ligase 2	HGNC:16804
	*GTF3C1*	5	transcription regulator	generaltranscription factor IIIC, polypeptide 1, alpha 220 kDa	HGNC:4664
	*MDN1*	5	nuclear chaperone	MDN1, midasin homolog (yeast)	HGNC:18302
	*DIDO1*	5	transcrption regulator	death inducer-obliterator 1	HGNC:2680
	*HIST1H3C*	5	histone protein	histone cluster 1, H3c	HGNC:4768
	*ANKRD13B*	4.9	unknown	ankyrin repeat domain 13B	HGNC:26363
	*CALCB*	4.9	hormone	calcitonin-related polypeptide beta	HGNC:1438
	*PTPRA*	4.9	phosphatase	protein tyrosine phosphatase, receptor type, A	HGNC:9664
	*STAT5A*	4.8	transcription regulator	signal transducer and activator of transcription 5A	HGNC:11366
	*LIMK1*	4.8	cytoskeleton	LIM domain kinase 1	HGNC:6613
	*SLC23A2*	4.7	transporter	solute carrier family 23 (nucleobase transporters), member 2	HGNC:10973
	*BARX1*	4.5	transcription regulator	BARX homeobox 1	HGNC:955
	*NHS*	4.5	unknown	Nance-Horan syndrome (congenital cataracts and dental anomalies)	HGNC:7820
	*MTAP*	4.5	glycosyltransferase	methylthioadenosine phosphorylase	HGNC:7413
	*FOXK1*	4.5	transcription regulator	forkhead box K1	HGNC:23480
	*PCMT1*	4.4	O-methyltransferase activity	protein-L-isoaspartate (D-aspartate) O-methyltransferase	HGNC:8728
	*SETD1A*	4.3	transcription regulator	SET domain containing 1A	HGNC:29010
	*CENPP*	4.2	centromere protein	centromere protein P	HGNC:32933
	*KIAA1217*	4.1	unknown	KIAA1217	HGNC:25428
	*SLITRK1*	4.1	enhances neuronal dendrite outgrowth	SLIT and NTRK-like family, member 1	HGNC:20297
	*RORB*	4	transcription regulator	RAR-related orphan receptor B	HGNC:10259
	*DLGAP1*	4	postsynaptic scaffold in neuronal cells	discs, large (Drosophila) homolog-associated protein 1	HGNC:2905
	*C3orf21*	4	unknown	chromosome 3 open reading frame 21	HGNC:26639
	*ST6GALNAC1*	4	protein glycosylation	ST6 (alpha-N-acetyl-neuraminyl-2,3-beta-galactosyl-1,3)-N-acetylgalactosaminide alpha-2,6-sialyltransferase 1	HGNC:23614
	*ZIC3*	3.9	transcription regulator	Zic family member 3 (odd-paired homolog, Drosophila)	HGNC:12874
	*LYPLA1*	3.8	hydrolase	lysophospholipase I	HGNC:6737
	*REST*	3.8	transcription regulator	RE1-silencingtranscription factor	HGNC:9966
	*TMEM132D*	-2	unknown	transmembrane protein 132D	HGNC:29411
	*MAP1LC3B2*	-2.1	microtubule	microtubule-associated protein 1 light chain 3 beta 2	HGNC:34390
	*CBY3*	-2.2	unknown	chibby homolog 3 (*Drosophila*)	HGNC:33278
	*RASIP1*	-2.3	signal transduction	Ras interacting protein 1	HGNC:24716
	*PRKG1*	-2.3	kinase	protein kinase, cGMP-dependent, type I	HGNC:9414
	*WDR72*	-2.4	unknown	WD repeat domain 72	HGNC:26790
	*KCNQ2*	-2.7	ion channel	potassium voltage-gated channel, KQT-like subfamily, member 2	HGNC:6296
	*BAIAP3*	-2.8	G-protein coupled receptor	BAI1-associated protein 3	HGNC:948
	*MAP3K10*	-30	kinase	mitogen-activated protein kinase kinase kinase 10	HGNC:6849
Unique for Adenocarcinomas	*MSC*	4.4	transcription regulator	musculin	HGNC:7321
	*FAM78B*	4.1	unknown	family with sequence similarity 78, member B	HGNC:13495
	*HOXA1*	3.6	transcription regulator	homeobox A1	HGNC:5099
	*SEPT9*	3.3	enzyme	septin 9	HGNC:7323
	*GAS1*	3.3	Cell cycle/growth	growth arrest-specific 1	HGNC:4165
	*PTPRN2*	3.1	phosphatase	protein tyrosine phosphatase, receptor type, N polypeptide 2	HGNC:9677
	*RSPO2*	2.7	Wnt receptor signaling pathway	R-spondin 2 homolog (*Xenopus laevis*)	HGNC:28583
	*POU3F3*	2.6	transcription regulator	POU class 3 homeobox 3	HGNC:9216
	*TRPA1*	2.5	transporter	transient receptor potential cation channel, subfamily A, member 1	HGNC:497
	*SYT6*	2.3	transporter	synaptotagmin VI	HGNC:18638
	*SLC6A2*	2.3	transporter	solute carrier family 6 (neurotransmitter transporter, noradrenalin), member 2	HGNC:11048
	*LHX1*	2.1	transcription regulator	LIM homeobox 1	HGNC:6593
	*RAPGEF5*	2.1	small GTPase mediated signal transduction	Rap guanine nucleotide exchange factor (GEF) 5	HGNC:16862
	*GDF10*	2.1	growth factor	growth differentiation factor 10	HGNC:4215
	*C3orf45*	1.6	unknown	chromosome 3 open reading frame 45	HGNC:26781
	*SLIT2*	1.6	differentiation/apoptosis	slit homolog 2 (Drosophila)	HGNC:11086
	*ZNF423*	−1.6	transcription regulator	zinc finger protein 423	HGNC:16762
	*RHOF*	−1.8	actin filament organization	ras homolog gene family, member F (in filopodia)	HGNC:15703

Genes were selected based on the average of log_2_ ratios from the samples in each group. Hypermethylated genes common in all tumors and unique for the ADC groups were selected based on log_2_ ratio > 2.5; hypomethylation included all genes from those groups. For SCC we selected the first 50 genes with the highest log_2_ ratios in hypermethylation, and log_2_ ratio < −2.0 for hypomethylated genes. Gene classes and their correspondent biological functions were retrieved from Gene Ontology (http://www.geneontology.org/) and Protein Knowledgebase UniProtKb (http://www.uniprot.org/).

## Methods

### Patient samples

Samples were obtained from the patients with NSCLC (n = 48) who had undergone surgical lung resection at Erasmus University Medical Center Rotterdam. Specimens were collected from the tumor and adjacent non-cancerous lung tissue and studied under an anonymous tissue protocol approved by the medical ethical committee of Erasmus University Medical Center Rotterdam. Tissues were snap-frozen within two hours after surgical resection in liquid nitrogen pre-cooled isopentane, and stored at 196 °C or 80 °C until DNA extraction.

### Histopathological analysis

Patient samples were independently reviewed by two pathologists. The cohort included 17 with ADC; 16 with SCC, seven with LCC , eight unclassified samples, and paired lung tissues for each tumor sample.

### Cell line

The MRC-5 lung fibroblast-like cell line was used as control. Cells were cultured under standard conditions using minimum essential medium supplemented with 10% heat-inactivated fetal bovine serum, 2 mM L-glutamine, 1% non-essential amino acids and penicillin/streptomycin. Cells were harvested when they reached 90% confluence.

### DNA isolation

Genomic DNA from patient tissues and cultured cells were extracted by overnight treatment incubation with lysis buffer and proteinase K, followed by phenol-chloroform extraction, ethanol precipitation and RNase digestion.

### Artificial demethylation and methylation of genomic DNA

We used DNA extracted from the MRC-5 cell line and commercially available Universal unmethylated DNA (UUD; Millipore(Billerica, MA, USA). Fully unmethylated DNA was obtained by whole-genome amplification using the REPLI-g kit (QiagenQiagen – Germantown, MD, USA) according to manufacturer protocol, followed by phenol-chloroform extraction. Fully methylated DNA was prepared by treating MRC-5 DNA and UUD with M.SssI enzyme (New England Biolabs - Ipswich, MA, USA) according to the manufacturer’s protocol. In short, 10 μg of DNA was incubated for 2 hours at 37°C with 40 U of M.SssI and 640 μM of S-adenosylmethionine. DNA was then treated with phenol-chloroform and recovered by ethanol precipitation.

### Methyl-DNA capture

Enrichment of methylated DNA was carried out using the MethylCap technique. DNA samples were sheared by sonication to obtain fragments between 200 and 800 bp. Methylated DNA capture was carried out using an adapted protocol from the MethylMiner Methylated DNA Enrichment kit (Invitrogen – Carlsbad, CA, USA). Two adaptations were made. Firstly, 20 μL of beads and 14 μL (7 μg) of MBD-biotin protein were used for 5 μg of sheared genomic DNA. Secondly, a single elution fraction was obtained by resuspending the beads in 200 μL of 1X binding/washing buffer containing 2 μL of proteinase K (20 mg/ml). The samples were then incubated for 90 minutes at 57 °C with 800 rpm agitation. Remaining steps were performed following the manufacturer’s protocol scaled up to 5 μg of DNA. Before the samples were used for high-throughput sequencing, methyl-enrichment was tested by quantitative (q) PCR (data not shown). qPCR was carried out using SYBR r green, Phire Hot Start DNA polymerase (Finnzymes - Vantaa, Finland)), and 1 μL of the enriched samples. Primer sequences are available in Additional file 16. qPCR was performed in triplicate and enrichment levels were calculated as previously described [50].

### High-throughput sequencing

High-throughput sequencing (HTS) was carried out on 18 samples: seven tumors (three ADC, three SCC and one LCC), seven paired lung tissues and four controls (two artificially fully methylated DNA and two fully unmethylated DNA). For each sample, DNA recovered from two independent Methyl Capture experiments was sequenced. The Illumina Genome Analyzer IIx platform (San Diego, CA, USA was used for both replicates, one sample per lane, according to the manufacturer’s protocol. In short: after MethylCap, fragments of the methyl-enriched fraction were end-repaired and ligated to single-read adaptors. Samples were size-selected to an average of 320 bp, PCR-amplified and 36 bp sequenced.

The Illumina Casava pipeline was used for base calling, alignment and quality control. Results were mapped against the reference Human_UCSChg18_AllChromosomes using eland_extended by Illumina pipeline 1.6.0.

### Bioinformatic analysis

Detailed information about the evaluation and selection of normalization parameters, and identification, characterization and selection of DMRs can be found in Additional file 3.

### Bisulfite sequencing

Bisulfite sequencing was carried out on the same 16 samples used for MethylCap HTS. Detailed information is provided in Additional file 3. In short: bisulfite conversion was carried out using the Epitect kit from Qiagen following the manufacturer’s protocol. Samples were amplified using primers specific for the fragments of interest ( Additional file 16). Amplified fragments were loaded on 2% agarose gels and extracted from the gel using a NucleoSpin Extract II kit (Machery-Nagel – Düren, Germany). Fragments were then ligated to pGEM-t easy vector (Promega - Madison, WI, USA) and cloned into DH12-β competent cells. Colony PCR was performed on 36 colonies and PCR fragments sequenced. Analysis of methylated and unmethylated CpGs was executed using two online programs: BISMA (http://biochem.jacobs-university.de/BDPC/BISMA) and QUMA (http://quma.cdb.riken.jp).

### Methylation-specific PCR

To determining the methylation status of the selected regions, we used MSP [33] in 96 samples (48 tumors and matching lung tissues). Samples were bisulfite converted as described for bisulfite sequencing, and amplified using two different set of primers designed for the methylated and unmethylated sequences ( Additional file 16). The fully methylated and fully unmethylated DNA samples were used as controls, and a water blank reaction used as control for contamination. After amplification, products were resolved on 2% agarose gels containing ethidium bromide and visualized under UV transillumination.

### Statistical analysis

Statistical analysis for bisulfite sequencing was provided by the BISMA program. In short, Fisher’s exact test was used for the statistical significance between two bisulfite sequence groups at each CpG, and a Mann-Whitney U-test for the statistical significance between two groups of the entire set of CpG sites. Two-tailed *P*-value of Fisher’s exact test was calculated from the 2 × 2 tables at each CpG site. A Mann-Whitney U-test was used for the statistical significance of the entire set of CpG sites between the tumor and lung groups. For determination of significance in the MSP, we used chi-square distribution. Two-tailed *P*-values were determined by the counts of the amplified bands for each primer in the group of samples lung/tumor or SCC/ADC. Groups that showed a *P*-value < 0.05 were considered significantly different.

## **Abbreviations**

ADC, adenocarcinoma; Bp, base pair; DMR, differentially methylated region; HTS, high-throughput sequencing; LCC, large cell carcinoma; LINE, long interspersed element; MBD, methyl-binding domain; MSP, methylation-specific PCR; NSCLC, non-small cell lung carcinoma; PCR, polymerase chain reaction; SCC, squamous cell carcinoma; UUD, Universal unmethylated DNA; UTR, untranslated region.

## Competing interests

The authors declare no that they have no competing interests.

## Authors’ contributions

RHC, VH, BL and SP participated in the concept and writing of the manuscript. RHC, WvI and CK executed specific experiments. RHC, VH, JH, ST, TvG, MvV, RB, ER, BL and SP were involved in the analysis of the data. RHC, VH, JA, BL and SP substantially contributed for interpretation of data. RHC, AS, JF and SP participated in the design of the experiments. RHC, JA, FG, and SP participated in editing or revising the manuscript. All authors have read and approved the final manuscript.

## Supplementary Material

Additional file 1 **TableS1.** Data from all samples used in this study. Click here for file

Additional file 2 **TableS2.** High-throughput sequencing reads from all samples and replicate experiments. Click here for file

Additional file 3 **Methods.** Detailed description of Bioinformatics approach and methods [51-55]. Click here for file

Additional file 4 **FigureS1.** Selection of normalization method. Distribution of Pearson’s correlation coefficients between replicates for different normalization methods. Click here for file

Additional file 5 **FigureS2.** Correlation between technical replicates. Scatter plots comparing raw MethylCap-seq signal from two technical replicates (R1 and R2) for all 14 samples. Each point represents one 10 bp genomic bin. Density of points (log10 scale) is shown in different shades of blue. Pearson’s correlation coefficient is denoted in the top left corner of each scatter plot. Click here for file

Additional file 6 **FigureS3.** Correlation between lung and tumor samples. Scatter plots comparing raw MethylCap-seq signal from healthy and tumor tissue for six pairs of samples. Each point represents one 10 bp genomic bin. Density of points (log10 scale) is shown in different shades of blue. Pearson’s correlation coefficient is denoted in the top left corner of each scatter plot. Click here for file

Additional file 7 **FigureS4.** Selection of *P*-value threshold. Number of significant hypo- and hypermethylated regions for different *P*-value thresholds. Y-axis shows number of significant regions on log10 scale for decreasing *P*-value threshold, shown on the x-axis. Click here for file

Additional file 8 **FigureS5.** along each chromosome for each paired sample. Representation of the log-ratio of relative methylation scores for tumors vs. paired lung samples for each sample pair showing DMRs along each chromosome. Green colors marks hypomethylation in tumor, negative log-ratio ≤2, and red colors marks hypermethylation in tumor, positive log-ration >2. Click here for file

Additional file 9 **TableS3.** Table with all DMRs found in this study. 14472 DMRs with chromosomal coordinates, log2 ratio, position where DMR is located regarding to genes and gene identities for DMRs that are in gene areas. Click here for file

Additional file 10 **FigureS6.** MethlCap-seq reads of two DMRs in all seven samples and replicate experiments. All 14 samples and two replicate experiments for two DMRs that were present in all tumors. (**A**) Sequence reads plotted in the UCSC genome browser spanning 100kbp in chromosome 2:63,090,001 to 63,190,000; (**B**) Sequence reads spanning 100 kbp in chromosome 14: 60,000,001 to 60,100,000. Reproducibility can be observed by the similarity between R1 and R2 of each sample. Red bar highlight hypermethylated DMRs in each chromosome found to be present in all seven tumor samples. Click here for file

Additional file 11 **FileS1.** Tables with hyper- and hypomethylated regions separated in “All tumors”, SCC and ADC subtypes. Tables show hyper- and hypomethylated regions found present in all tumors or that were either unique for SCC or ADC, separated per tumor subtype and DMR type. Click here for file

Additional file 12 **FigureS7.** Methylation status of CpGs per paired sample in all five fragments. **A**) Model of analysis. Blue = unmethylated; red = methylated. CpGs are horizontally ordered and clones vertically ordered. Per paired samples in each fragment, the average of methylation of each CpG is calculated and represented from blue to red depending on the percentage of clones that were methylated in the specific CpG. Top panel represents lung samples, bottom panel: tumor samples. **B**) All paired analyses for CDKN2A, RASSF1, Frag_01, Frag_02 and EN1. Click here for file

Additional file 13 **FileS2.** Tables with bisulfite sequence analysis of tumor versus lung. Counts and statistics per CpG for all clones in CDKN2A, RASSF1, Frag_01, Frag_02 and EN1 fragments. Click here for file

Additional file 14 **FileS3.** Tables with bisulfite sequencing statistic analysis of SCC versus ADC. Statistics per CpG for all pooled SCC samples versus pooled ADC samples. Click here for file

Additional file 15 **FigureS8.** MSP screening. Cropped gel images for all 48 samples grouped by histological subtype in the five fragments analyzed: 17 ADC, 16 SCC, 7 LCC, 8 others (other types and unclassified NSCLC) M: primer for methylated CpG; N: normal/healthy; T: tumor; *: samples used in MethylCap-seq; TM: Totally methylated control sample; TU: Totally unmethylated control sample; ø: blank/water; U: primer for umethylated CpG. Click here for file

Additional file 16 **TableS4.** Primers. Sequence and conditions of primers used for Bisulfite sequencing and MSP. Click here for file
