# Supplementary material for: Genome-wide DNA methylation profiling of non-small cell lung carcinomas
Source: Epigenetics Chromatin. 2012 Jun 22;5:9. doi: 10.1186/1756-8935-5-9 (PMC3407794; doi:10.1186/1756-8935-5-9)
Supplement: Additional file 3 — Methods. Detailed description of Bioinformatics approach and methods [51-55]. [file 1756-8935-5-9-S3.doc]

**Additional File 03 – Detailed description of Bioinformatics approach and methods**

**Bioinformatics analysis**

Processing of raw mapped reads and all downstream analyses were done using the R statistical computing environment, version 2.12.0 (<http://www.r-project.org/>) [51] and specialized software packages within the Bioconductor project [52] (<http://www.bioconductor.org/>). Total number and quality of sequenced reads per DNA sample is summarized in Supplementary Table 2. We selected uniquely mapped reads with quality score $\geq$ 15 and generated genome-wide coverage by extending reads up to 200 bp. Raw coverage was visualized in the ([http://genome.ucsc.edu/](http://genome.ucsc.edu/index.html)) [53] in the context of the human genome (International Human Genome Sequencing Consortium, 2001), March 2006 assembly (hg18).

***MethylCap-seq data normalization***

For normalization of the MethylCap-seq data we adopted the CpG coupling factor–based normalization method from [39] with certain modifications. It has been shown that MeDIP-derived data shows bias that depends on local CpG densities which is caused by a varying efficiency of antibody binding and immunoprecipitation [54-55]. MethylCap experiments that use methyl-binding protein domains (MBD) instead of antibodies follow a similar experimental procedure and thus may encounter the same problem. To compute unbiased methylation signals, raw data has to be normalized with respect to local CpG density. Our normalization approach is based on the concept of coupling factors presented by Down et al. (2008). We combined modeling of dependency between increasing total CpG density and increasing mean MethylCap-seq signal proposed by Chavez et al. (2010) with using an artificially fully methylated reference sample to calculate normalization parameters.

The MEDIPS software package [39] was used to generate raw short reads coverage at 10 bp resolution. Corresponding coupling factors (CF) were calculated by counting number of CpG dinucleotides in varying region widths (+/- 200, 300, 500 and 700 bp) around each 10 bp genomic bin. For the full range of CF values we calculated the mean raw methylation signal of all bins associated with a given value of CF and used it to generate a calibration curve.

We tried several different approaches for modeling dependency between local CpG density and MethylCap-seq signal using varying region widths for coupling factor calculation and fitting two different types of models. First we employed the original method proposed by Chavez et al. (2010) that fits a linear model on the initial portion of the calibration curve, which corresponds to low CF values. This approach is based on the assumption that regions in the genome with low CpG densities are in general fully methylated. Normalization parameters calculated in this way are represented by slope and intercept that are calculated for each sample separately depending on the corresponding calibration curve. However, such a model is derived from a relatively small range of dependency of signal on CpG density that is in real DNA samples limited to lower CF values. Since we conducted a reference experiment using an artificially fully methylated DNA sample we were able to get the real dependency of signal on CpG density over a much larger range of CF values. Therefore, by fitting a model on a calibration curve derived from the fully methylated DNA sample we include a much larger proportion of the data (~ 70%) and the calculated parameters are more reliable. In addition, we tried to fit a logistic model instead of the linear model since it captures much better the trend of saturation that is expected to occur for regions with high CpG density. Although we see the saturation in our fully methylated sample, it is limited to a relatively low range of CF values after which the mean signal drops again, which we assume is due to lower efficiency of artificial methylation for the regions with very high CpG density and the much lower number of fragments with high CpG density available during sequencing.

To select the best performing normalization method we evaluated the resulting normalized signal in two independent ways. Since we had technical replicates for all our samples, this enabled us to monitor the correlation of normalized signal between replicates. In addition, the bisulfite sequencing experiments for 5 selected genomic regions that we carried out to validate the MethylCap-seq results, enabled us to monitor the correlation of normalized signal and actual percentage of methylated CpG dinucleotides in given regions. Results of the evaluation of different normalization parameters are summarized in Supplementary Figure 1. They show that using parameters obtained by fitting a logistic model to the fully methylated sample for normalizing all other samples preserves correlation between technical replicates and in addition keeps a good correlation to the results of bisulphate sequencing. The logistic model fitted to the calibration curve for the fully methylated sample can be described by the following equation:

$$m_{CF}=\frac{Asym}{1+e^{(\frac{xmid-CF}{scal})}}$$

where *CF* is the coupling factor, *Asym*, *xmid* and *scal* are estimated parameters and *m* is the predicted value of methylation signal in the 10bp genomic bin having a given value of coupling factor.

The obtained parameters (*Asym*, *xmid*, *scal*) were used to normalize raw methylation signals for each sample as follows:

$$rms_{i}=\frac{m_{i}}{\frac{nreads_{sample}}{nreads_{reference}}*\frac{Asym}{1+e^{(\frac{xmid-CF_{i}}{scal})}}}$$

where *m_i_* is the raw methylation signal of an individual 10pb genomic bin, *CF_i_* is the corresponding value of coupling factor, *nreads_reference_* is the total number of sequenced reads for artificially fully methylated reference sample and *nreads_sample_* is the total number of sequenced reads for a given sample. Calculated relative methylation scores (*rms*) can be used for comparison of methylation signal across different samples (e.g. detection of differential methylation) since they are corrected for the total number of reads per sample.

***Genome-wide methylation profiles: Identification and characterization of DMRs***

Two technical replicates for each sample were merged by calculating mean *rms* of replicates for each 10bp bin. Next we calculated mean *rms* values for genome-wide non-overlapping 500 bp windows for all 14 samples to generate genome-wide methylation profiles. Global background signal threshold was estimated from the fully unmethylated DNA sample used as a background by selecting 90^th^ percentile of *rpm* (reads per million) values for all 500bp genome-wide windows. Regions with either *rpm* value below the selected threshold or with *rpm* ratio $\frac{rpm_{sample}}{rpm_{input}}$ < 1.33 for both healthy and tumor sample were excluded. Finally, we tested remaining regions for differential methylation between corresponding tumor and healthy samples using the MEDIPS software package [39]. Distributions of 10bp bins *rms* values within each 500bp genomic region were compared using both one-sided Student’s t-test and one-sided Wilcoxon rank-sum test. Testing was done separately for hypo- and hypermethylation and p-value threshold of 10^-18^ (corresponding to a false discovery rate *≤ 10^-15^* upon correction for multiple testing) was used to select significant DMRs.

For each sample, regions with p-value $\leq$ 10^-18^ (FDR *≤ 10^-15^*) were projected onto the genome and visualized as genome-wide maps of differential methylation. Composition of DMRs was calculated on a set of top 5000 hypomethylated and top 5000 hypermethylated regions with lowest p-values as a percentage of regions covered by selected genomic features. We retrieved annotation for human transcripts from Ensembl (V60) (<http://nov2010.archive.ensembl.org/index.html>) and annotation for repeats from UCSC Genome Browser database [53]. Non-overlapping sets of distinct classes of repeats were created and these regions were excluded from subsequent genomic features. All Ensembl transcripts were used to generate non-overlapping sets of promoters (region +/- 1 kb around TSS), exons and introns.

***Cluster analysis, identification and characterization of common DMRs and associated genes***

We created a non-redundant set of genomic regions by combining DMRs with p-value $\leq$ 10^-18^ from all 7 samples. Feature matrix containing $\log_{2}(\frac{rms_{tumor}}{rms_{healthy}})$ for all 7 samples at all selected regions was used to calculate Manhattan distance between samples as well as between selected regions. We performed hierarchical clustering on obtained dissimilarity matrices and visualized the result as a heatmap. Clusters of DMRs were definded by cutting clustering dendrogram at height=23 and were projected onto the genome to visualize their genomic localization. To measure the enrichment of DMRs in the subtelomeric area, we calculated for each chromosome arm separately the ratio between proportion of 1Mb region at chromosome end and proportion of the entire chromosome covered by DMRs. We calculated analogous ratios for randomly sampled regions and compared them to DMR’s using Student’s t-test. In addition, DMRs were overlapped against the set of all Ensembl transcripts to reveal the association to distinct parts of genes (promoters: +/- 1 kb around TSS, three prime end: +/- 1kb around TTS, gene body) and percentages of regions within each cluster associated to distinct gene parts was calculated. We identified DMRs common to all samples (or a subset of samples) by selecting regions with |$\log_{2}(\frac{rms_{tumor}}{rms_{healthy}})$| $\geq$ 1 (two-fold change in methylation signal) for all corresponding samples.

***Bisulfite Sequencing***

Primers for bisulfite sequencing were designed using a combination of tools, such as MethPrimer (<http://www.urogene.org/methprimer/index1.html>), Methyl Primer express software v.1.0 from Applied Biosystems, and manual selection (supplementary table 4). Amplification conditions were as follows: 1X PCR buffer (Invitrogen); 200 µM of dNTP; 1.5 mM of MgCl_2_; 0.2 µM of primers; 0.2 µl of Platinum Taq (Invitrogen) and 2 µl of the bisulfite treated sample. Amplification was carried out in 50 µl using the same conditions as described above. Colony PCR protocol was as follows: 94 ˚C for 5 min, 30 cycles of 94 ˚C for 15s, 55 ˚C for 30s, 72 ˚C for 30 sec, finalized by 72 ˚C for 5 min. PCR products were recovered using NucleoFast 96 PCR clean-up plates (Machery-Nagel), according to manufacturer instructions, and sequenced using T7 primer and a 3730 DNA Analyzer (Applied-Biosystems).

***Methylation-specific PCR (MSP)***

Samples were amplified in 50 µl reaction volume containing 1X PCR buffer (Invitrogen); 200 µM of dNTP; 1,5 mM of MgCl_2_; 0,2 µM of each primer; 0,2 µl of Platinum Taq (Invitrogen) and 1 µl of bisulfite treated sample. PCR was hot-started at 95 ˚C for 5 min, with 30 cycles of 95 ˚C for 30s, specific primer annealing temperature for 30s and 72˚C for 30 sec, finalized by 72˚C for 5 min. Primer sequences, annealing temperatures and product sizes and described in supplementary table 4.
