# Supplementary figures and images for: Genome-wide DNA methylation profiling of non-small cell lung carcinomas
Source: Epigenetics Chromatin. 2012 Jun 22;5:9. doi: 10.1186/1756-8935-5-9 (PMC3407794; doi:10.1186/1756-8935-5-9)

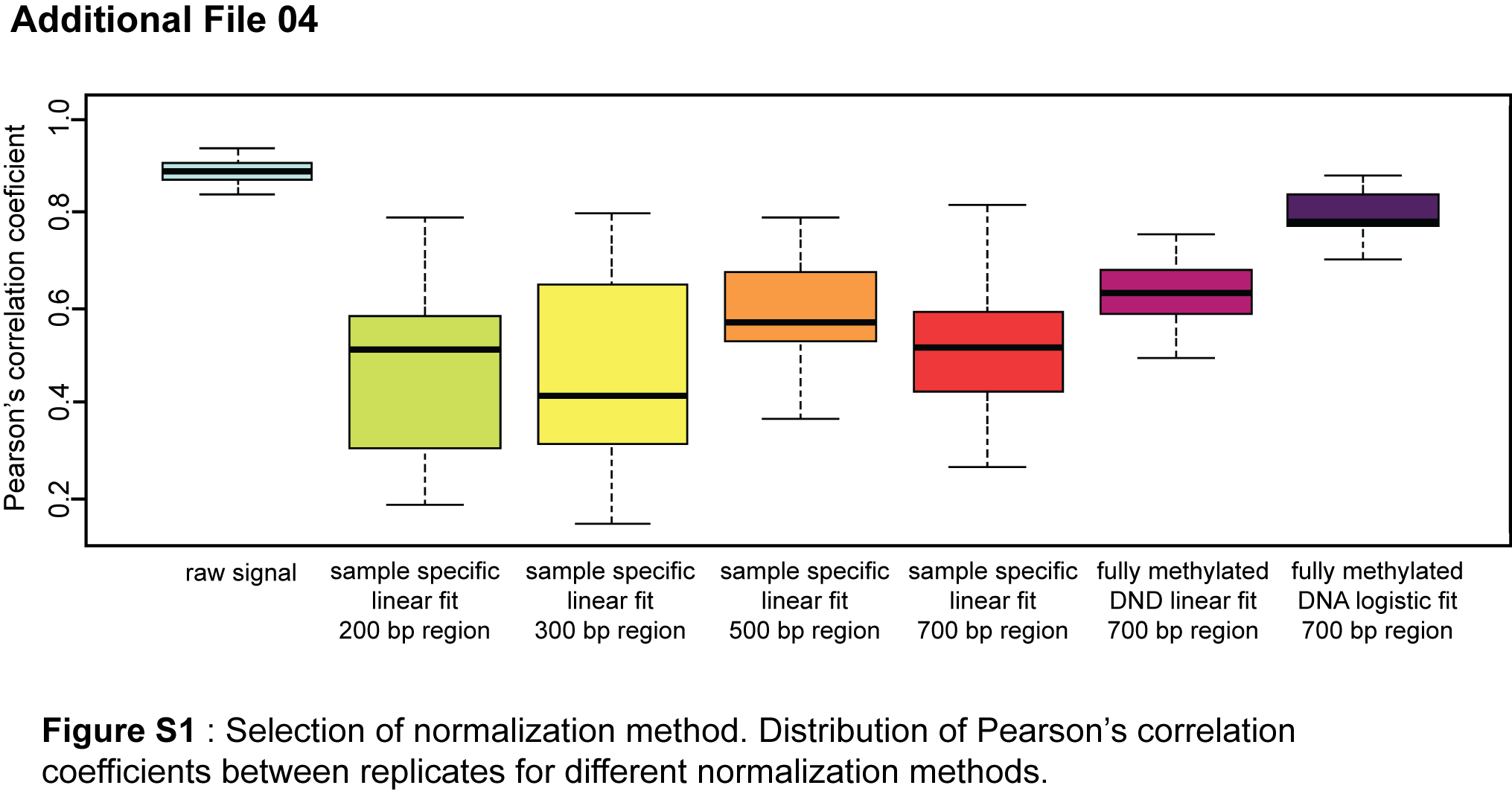

Supplement: Additional file 4 — FigureS1. Selection of normalization method. Distribution of Pearson’s correlation coefficients between replicates for different normalization methods. [file 1756-8935-5-9-S4.png]

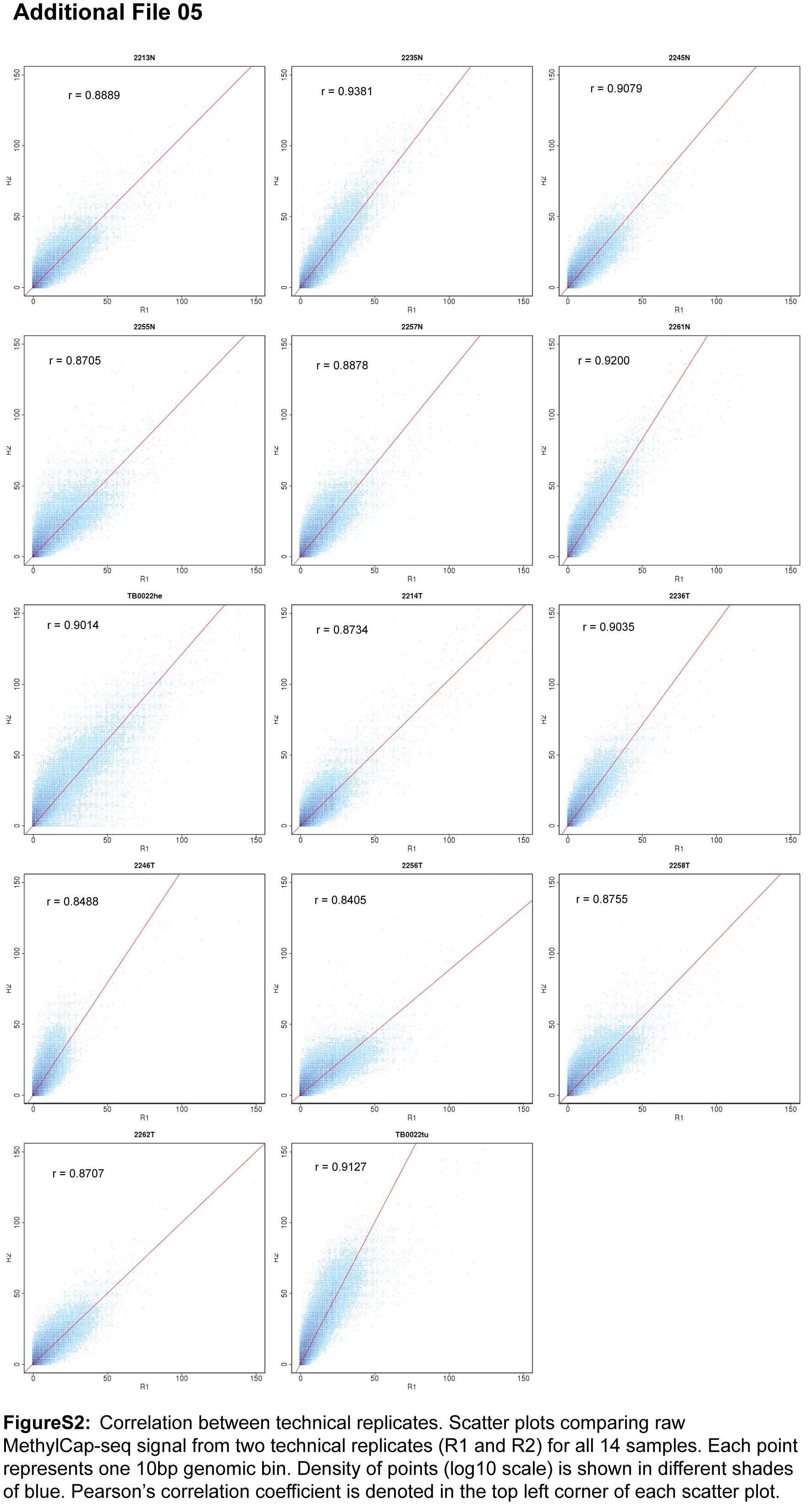

Supplement: Additional file 5 — FigureS2. Correlation between technical replicates. Scatter plots comparing raw MethylCap-seq signal from two technical replicates (R1 and R2) for all 14 samples. Each point represents one 10 bp genomic bin. Density of points (log10 scale) is shown in different shades of blue. Pearson’s correlation coefficient is denoted in the top left corner of each scatter plot. [file 1756-8935-5-9-S5.png]

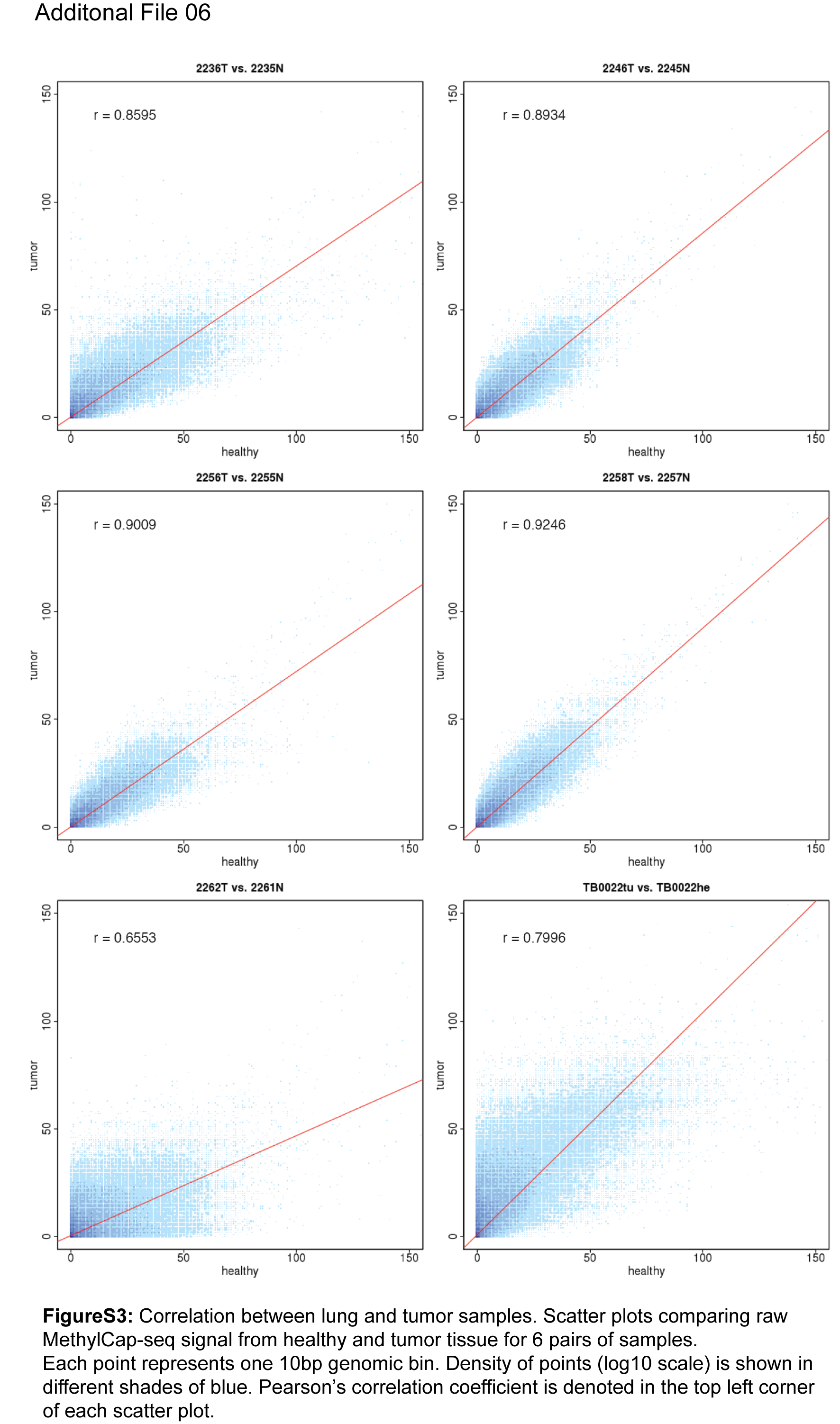

Supplement: Additional file 6 — FigureS3. Correlation between lung and tumor samples. Scatter plots comparing raw MethylCap-seq signal from healthy and tumor tissue for six pairs of samples. Each point represents one 10 bp genomic bin. Density of points (log10 scale) is shown in different shades of blue. Pearson’s correlation coefficient is denoted in the top left corner of each scatter plot. [file 1756-8935-5-9-S6.png]

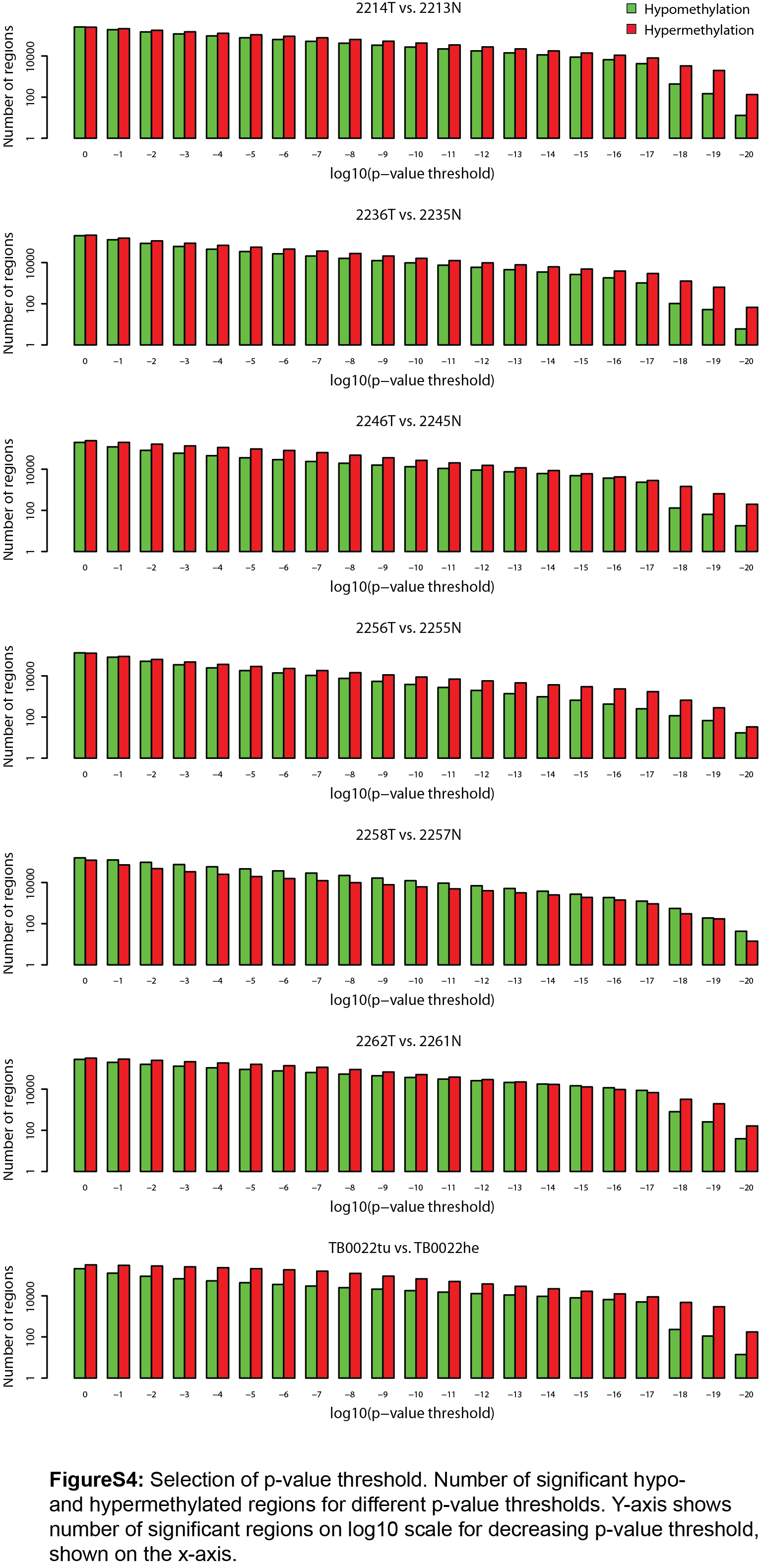

Supplement: Additional file 7 — FigureS4. Selection of P-value threshold. Number of significant hypo- and hypermethylated regions for different P-value thresholds. Y-axis shows number of significant regions on log10 scale for decreasing P-value threshold, shown on the x-axis. [file 1756-8935-5-9-S7.png]

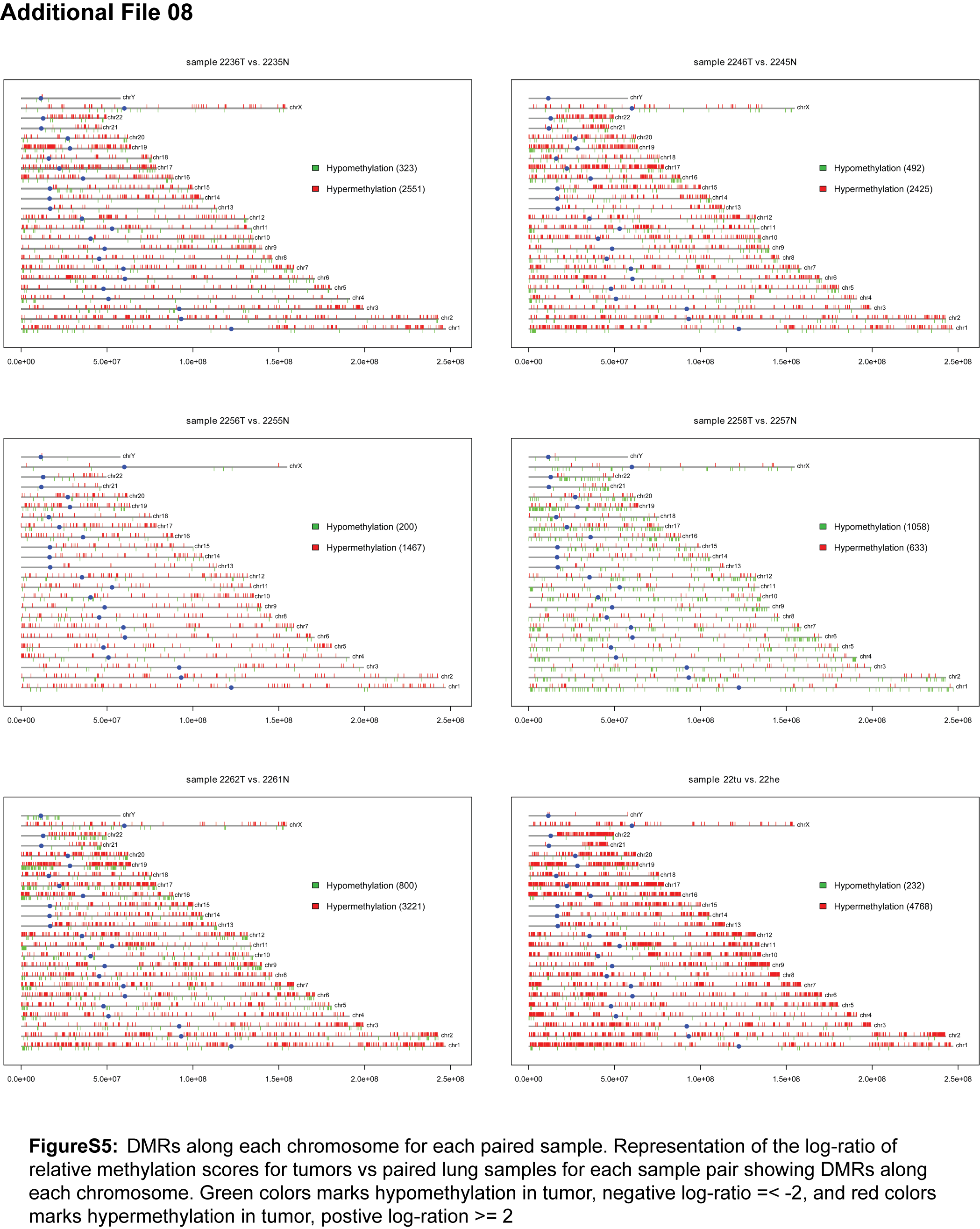

Supplement: Additional file 8 — FigureS5. along each chromosome for each paired sample. Representation of the log-ratio of relative methylation scores for tumors vs. paired lung samples for each sample pair showing DMRs along each chromosome. Green colors marks hypomethylation in tumor, negative log-ratio ≤2, and red colors marks hypermethylation in tumor, positive log-ration >2. [file 1756-8935-5-9-S8.png]

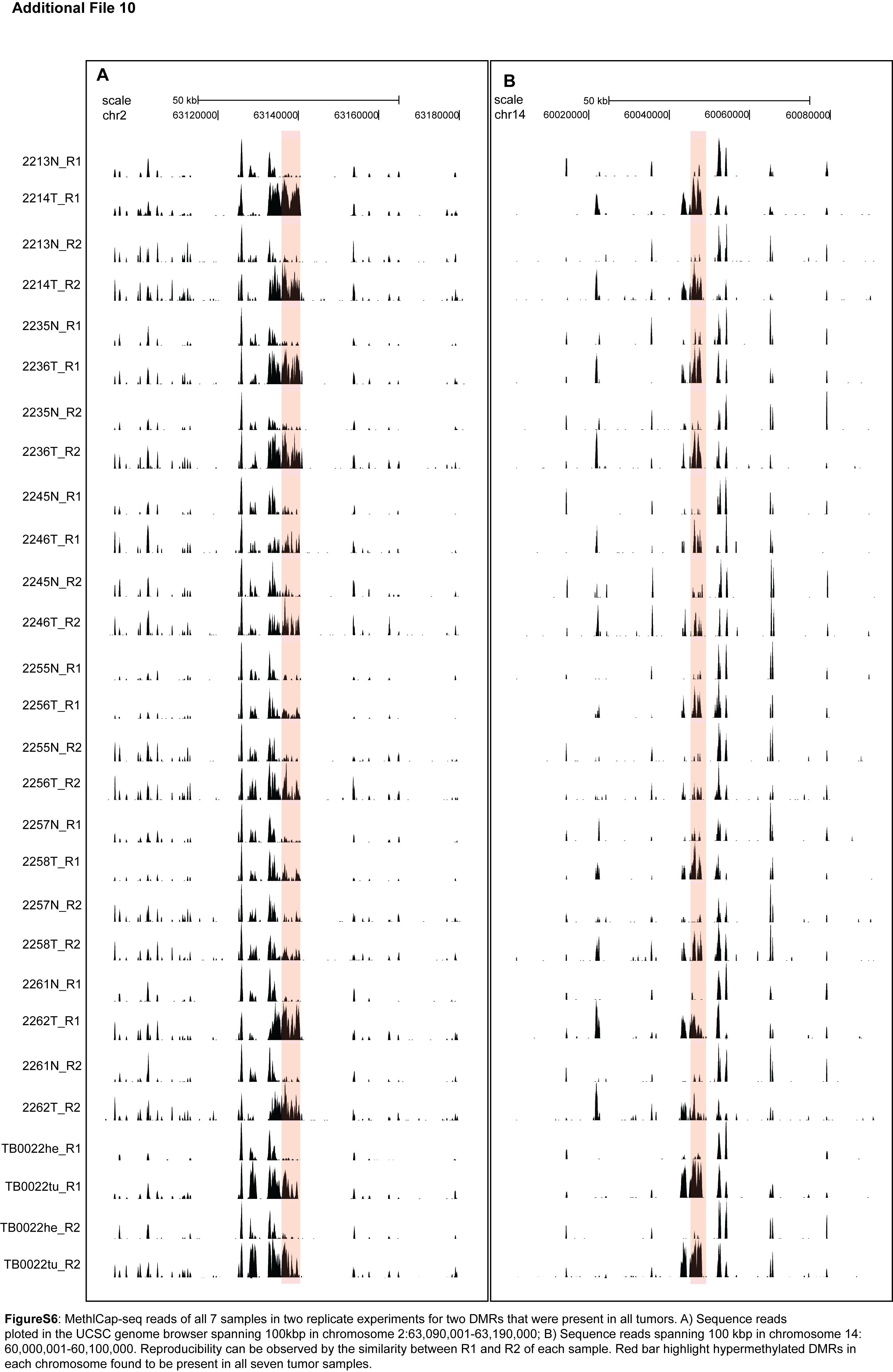

Supplement: Additional file 10 — FigureS6. MethlCap-seq reads of two DMRs in all seven samples and replicate experiments. All 14 samples and two replicate experiments for two DMRs that were present in all tumors. (A) Sequence reads plotted in the UCSC genome browser spanning 100kbp in chromosome 2:63,090,001 to 63,190,000; (B) Sequence reads spanning 100 kbp in chromosome 14: 60,000,001 to 60,100,000. Reproducibility can be observed by the similarity between R1 and R2 of each sample. Red bar highlight hypermethylated DMRs in each chromosome found to be present in all seven tumor samples. [file 1756-8935-5-9-S10.png]

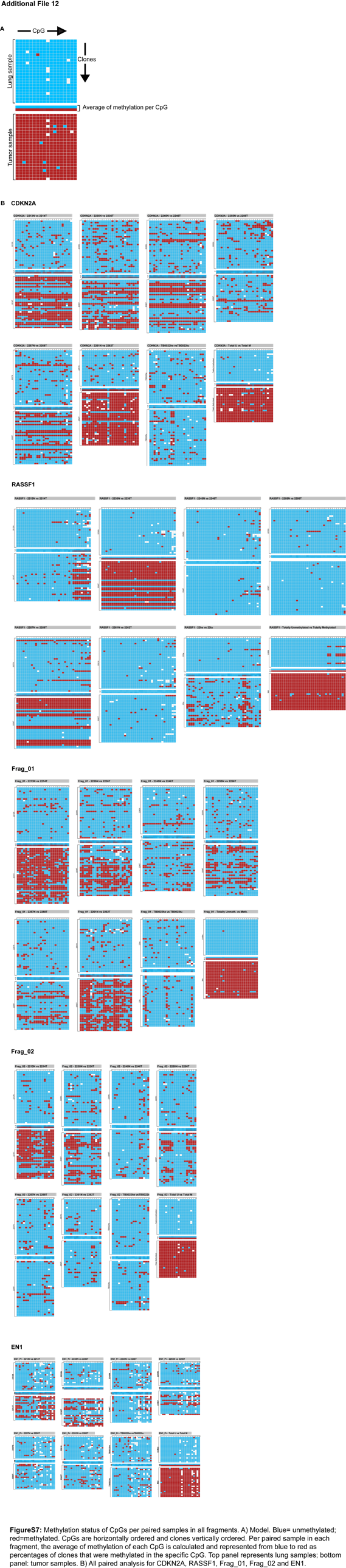

Supplement: Additional file 12 — FigureS7. Methylation status of CpGs per paired sample in all five fragments. A) Model of analysis. Blue = unmethylated; red = methylated. CpGs are horizontally ordered and clones vertically ordered. Per paired samples in each fragment, the average of methylation of each CpG is calculated and represented from blue to red depending on the percentage of clones that were methylated in the specific CpG. Top panel represents lung samples, bottom panel: tumor samples. B) All paired analyses for CDKN2A, RASSF1, Frag_01, Frag_02 and EN1. [file 1756-8935-5-9-S12.png]

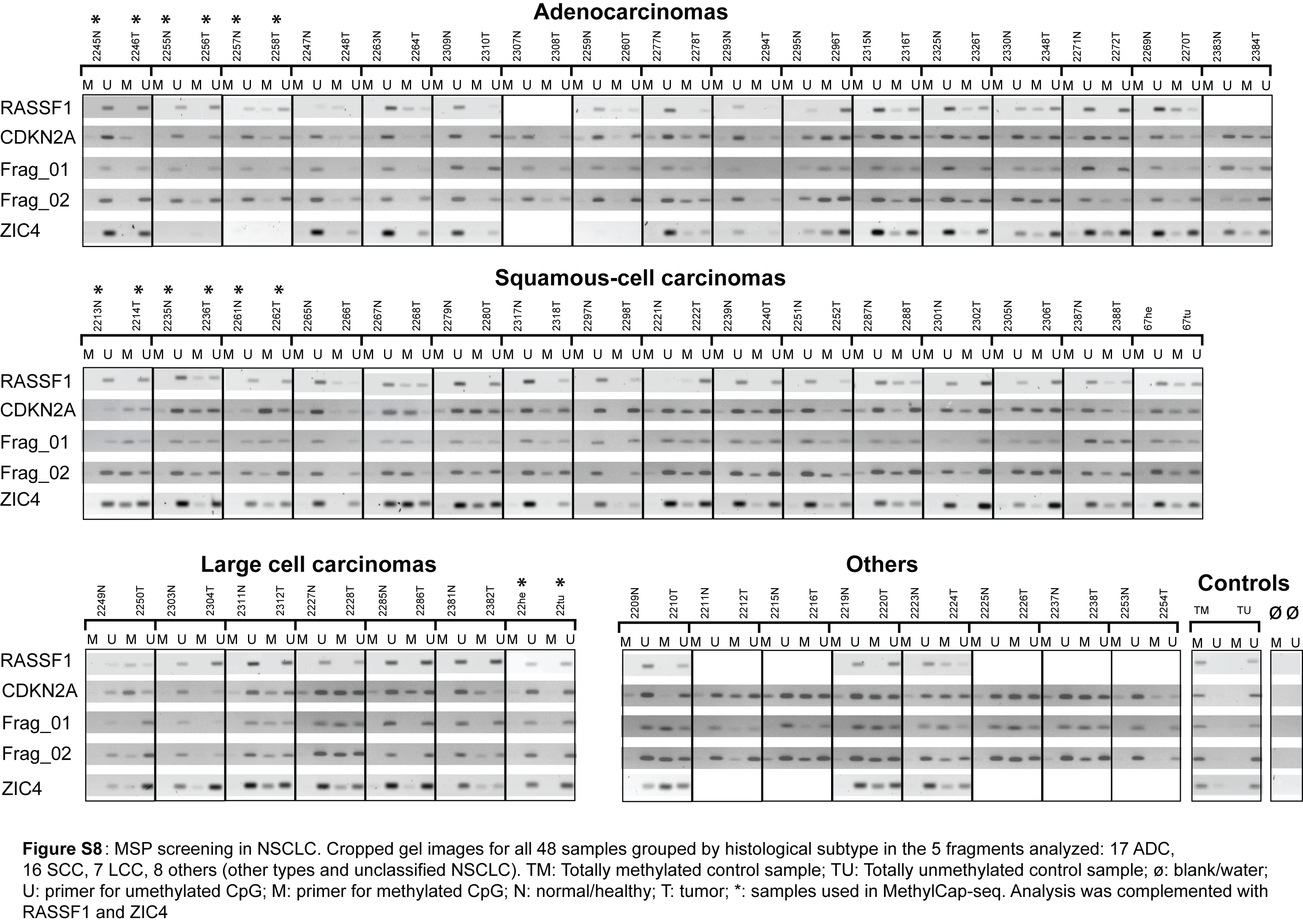

Supplement: Additional file 15 — FigureS8. MSP screening. Cropped gel images for all 48 samples grouped by histological subtype in the five fragments analyzed: 17 ADC, 16 SCC, 7 LCC, 8 others (other types and unclassified NSCLC) M: primer for methylated CpG; N: normal/healthy; T: tumor; *: samples used in MethylCap-seq; TM: Totally methylated control sample; TU: Totally unmethylated control sample; ø: blank/water; U: primer for umethylated CpG. [file 1756-8935-5-9-S15.png]
